# Physical activity in established rheumatoid arthritis and variables associated with maintenance of physical activity over a seven-year period – a longitudinal observational study

**DOI:** 10.1186/s41927-020-00151-6

**Published:** 2020-10-08

**Authors:** Ann Bremander, Karina Malm, Maria L. Andersson, on behalf of the BARFOT study group

**Affiliations:** 1 Department of Clinical Sciences, Rheumatology, Faculty of Medicine, Lund University, Lund, Sweden; 2 Department of Regional Health Research, University of Southern Denmark, Odense, Denmark; 3 Danish Hospital for Rheumatic Diseases, University Hospital of Southern Denmark, Sønderborg, Denmark; 4 Research and Development Center, Spenshult, Halmstad, Sweden; 5 Rheumatology, Capio Movement, Halmstad, Sweden

**Keywords:** Rheumatoid arthritis, Physical activity, Exercise, Longitudinal study

## Abstract

**Background:**

A large number of patients with RA do not adhere to the recommended levels of physical activity to enhance health. According to EULAR recommendations, physical activity should be part of standard care in people with rheumatic diseases. There have been few larger studies on maintenance of physical activity over longer periods of time. The aim was to study self-reported physical activity levels over 7 years in patients with established rheumatoid arthritis (RA). In addition, to determine variables associated with maintenance or change of physical activity behavior.

**Methods:**

Questionnaires were sent to the BARFOT cohort in 2010 (*n* = 1525) and in 2017 (*n* = 1046), and 950 patients responded to both questionnaires. Patients were dichotomized according to meeting MVPA recommendations (physically active at a moderate level ≥ 150 min/week or at an intense level ≥ 75 min/week) or not. Body mass index, smoking habits, tender joint count (TJC), swollen joint count (SJC), Patient Global Assessment (PatGA), pain intensity and distribution, fatigue, physical function (HAQ), health-related quality of life (EQ. 5D), comorbidities, and medical treatment were assessed. We used logistic regression analysis to study variables associated with maintenance and/or change of MVPA behavior.

**Results:**

Forty-one per cent (*n* = 389) of the patients met MVPA recommendations on both occasions. Patients who met MVPA recommendations over 7 years were younger and a higher proportion were never-smokers. There was a negative association with being overweight or obese, having cardiovascular or pulmonary diseases, pain, fatigue, and physical function, whereas there was a positive association between QoL and maintaining MVPA recommendations. Similar factors were positively associated with a deterioration in physical activity level over time.

**Conclusions:**

Maintenance of physical activity over a long period of time is challenging for patients with established RA. Reports of high quality of life supported maintenance of physical activity while disease related and unhealthy lifestyle factors had a negative effect. Health professionals should consider the patient’s standpoint when encouraging maintenance of physical activity, preferably using coordinated lifestyle interventions.

## Background

Promoting a healthy lifestyle has been popular in the past decade, not only in the general population [1, 2] but also for people with chronic inflammatory diseases. Interventions to support changes in lifestyle such as healthy eating and drinking, smoking cessation, and increasing the amount of weekly physical activity and exercise have been recommended in a number of guidelines to promote health in the rheumatoid arthritis (RA) population [3, 4]. Patients with RA have a higher risk of developing cardiovascular diseases and metabolic syndrome, among other comorbidities [5, 6]. Physical activity reduces the risk of these comorbidities in the general population [7], and there is some evidence that it might also be an effective treatment for patients with RA [8, 9]. It is well known that a large number of patients with RA have a sedentary lifestyle and are less active than their healthy counterparts [10]. Large population-based studies have found a prevalence of health-enhancing physical activity ranging from 20 to 70% in people with chronic inflammatory arthritis [11–13], depending on the methodology used and the country investigated.

Recommendations concerning health-enhancing physical activity require moderate physical activity (MPA) for ≥150 min/week or PA at a vigorous level for ≥75 min/week (VPA) [14, 15]. These recommendations also apply to people with rheumatic diseases [9]. Being physically active, defined as meeting moderate-to-vigorous physical activity (MVPA) recommendations, has the possibility of reducing disease burden in RA and may contribute to an improved quality of life (QoL) [12]. Factors that negatively affect the possibility of being physically active according to the recommendations are higher disease activity, worse pain and function, lower mental health, older age, worse health status, lower education, lower self-efficacy, and lack of social support [16–18]. While the prevalence of meeting physical activity recommendations has been well studied, there have been few larger studies on maintenance over longer periods of time [16, 18]. Maintaining a physically active lifestyle will affect overall health and may improve the impact of some common comorbidities in the RA population. A better understanding of variables associated with maintenance, improvement or deterioration in physical activity levels over time can help health care professionals to coach patients with RA in behavior change. The aim of the present study was to investigate self-reported physical activity levels over 7 years in a well-defined RA cohort, and to determine variables associated with maintenance or change of physical activity behavior.

## Methods

### Patients

This study involved patients with RA from the BARFOT (Better Anti-Rheumatic PharmacOTherapy) cohort, who were recruited between 1992 and 2006 and included in the study at the time for diagnosis (*n* = 2837). All patients fulfilled the American College of Rheumatology (ACR) criteria for classification of RA [19] and had a disease duration of ≤12 months. The patients were treated with DMARDs in accordance with the recommended treatment strategy in Sweden, as described in earlier studies [20]. The patients were assessed according to a structured protocol at inclusion and after 3, 6, 12, 24, 60, 96, and 180 months. In 2010 and in 2017, a lifestyle questionnaire was sent to the patients. There were response rates of 73% (*n* = 1525) and 68% (*n* = 1046) respectively, and 950 patients responded to both questionnaires. These patients made up our study cohort.

### Lifestyle questionnaire

In this study, well-used and validated outcome measures hypothesized to be of importance for patient-reported physical activity were assessed: disease characteristics, measures of health-related quality of life, physical functioning, fatigue, intensity and distribution of pain, and additional lifestyle information such as smoking and body mass index (BMI). The data were self-reported and retrieved from the 2010 questionnaire.

Disease characteristics assessed were self-reported tender joint count (TJC) and swollen joint count (SJC, 28 joints) [21], medical treatment, and comorbidity. Health-related quality of life was measured with the Euroqol 5 Dimensions (EQ-5D; 0–1, worst to best) [22] and patient global assessment (PatGA; numeric rating scale (NRS) 0–10, best to worst) [21]. Physical functioning was assessed with the Health Assessment Questionnaire (HAQ; 0–3, best to worst) [23]. Fatigue (NRS 0–10, best to worst), pain intensity (NRS 0–10, best to worst), and pain distribution (pain mannequin, with 18 defined body regions) were assessed [24].

Physical activity was assessed with questions concerning frequency and duration, reported as minutes/week spent on moderate physically activity and on vigorous physical activity. The same two questions were posed in 2010 and in 2017, and adhered to Swedish population studies on lifestyle habits (only available in Swedish, https://www.socialstyrelsen.se/globalassets/sharepoint-dokument/dokument-webb/nationella-riktlinjer/levnadsvanor-fragor-om-levnadsvanor.pdf). To assess maintenance and change of physical activity levels, data from the questionnaires were dichotomized as either being active at recommended levels of physical activity for enhancement of health (MVPA: physically active at a moderate level ≥ 150 min/week (MPA) or at a vigorous level ≥ 75 min/week (VPA)) or not (non-MVPA) [25].

### Statistics

To test differences between groups, the chi-square test was used for proportions and Student’s independent t-test was used for continuous variables, and all tests for significance were 2-tailed. The study sample was dichotomized as either achieving recommended levels of physical activity (MVPA) in 2010 and/or in 2017 or not (dependent variable). Three analyses were performed, (i) patients who met MVPA recommendations at both time points vs. participants who did not meet MVPA in 2010 or in 2017, (ii) patients who met MVPA recommendations at both time points vs. participants who reported a deterioration in physical activity level between 2010 and 2017, and (iii) patients who reported an improvement in physical activity level between 2010 and 2017 vs. patients who did not meet MVPA recommendations at any time point.

In this longitudinal study, we focused on variables from the 2010 questionnaire that were associated with the outcome 7 years later. Logistic regression analyses were used. All variables were adjusted for age, gender, and smoking habits. Statistical analyses were performed using SPSS Statistics 21 software (IBM Corp., Armonk, NY, USA).

## Results

Altogether, 950 patients responded to both questionnaires; mean age was 61 (SD 13) years with a mean disease duration of 9 (SD 4) years in 2010, and 72% were women (Table 1). The patients who were lost to follow-up were at inclusion in the BARFOT cohort, older (mean age 58 (SD 16) years vs. 52 (SD 13) years; *p* < 0.001), had a worse DAS28 (mean 5.34 (SD 1.23) vs. 5.12 (SD 1.24); *p* < 0.001), and a worse HAQ (mean 1.02 (SD 0.63) vs. 0.96 (SD 0.59); *p* = 0.024).

**Table 1:** Descriptive of all patients who answered the questionnaire in 2010 and 2017

		All patients in 2010 Mean (SD)	All patients in 2017 Mean (SD)
N		950	950
Age		61 (13)	
gender	Women, %	72	
Smoking habits	Non smoker, %	42	42
	Smoker, %	16	11
	Previous smoker,	42	47
RF, %		66	
Disease duration, years		8.7 (3.8)	
BMI		25.9 (4.2)	25.9 (4.8)
BMI	< 18.5, %	1	1
	18.5–24.9, %	44	45
	25.0–29.9, %	41	38
	≥30, %	14	15
Fulfilling MVPA, %		66	53
TJC		5.4 (6.3)	5.1 (6.4)
SJC		3.5 (5.1)	2.9 (4.8)
PatGA NRS (0–10)		2.9 (2.3)	3.0 (2.4)
Pain NRS (0–10)		3.3 (2.5)	3.4 (2.5)
Fatigue NRS (0–10)		4.1 (2.8)	4.1 (2.8)
HAQ		0.52 (0.55)	0.60 (0.62)
EQ 5D		0.74 (0.20)	0.72 (0.24)
Tender regions		4.3 (3.8)	3.9 (3.7)
Cardiovascular disease, %		41	50
Pulmonary disease, %		9	12
DMARD	No DMARD, %	17	24
	cDMARD^a^	54	43
	bDMARD^a^	26	27
	Only corticosteroids^a^	3	6

^a^with and without corticosteroids

*BMI* Body mass index, *TJC* Tender joint count, *SJC* Swollen joint count, *PatGA* Patient Global Assessment, *NRS* Numeric rating scale, *HAQ* Health Assessment Questionnaire, *EQ. 5D* Euroqol 5 Dimensions, *DMARDs* Disease modifying anti-rheumatic drugs, *cDMARDs* Conventional DMARDs, *CS* Corticosteroids

### Physical activity levels over seven years

**Fig. 1:**
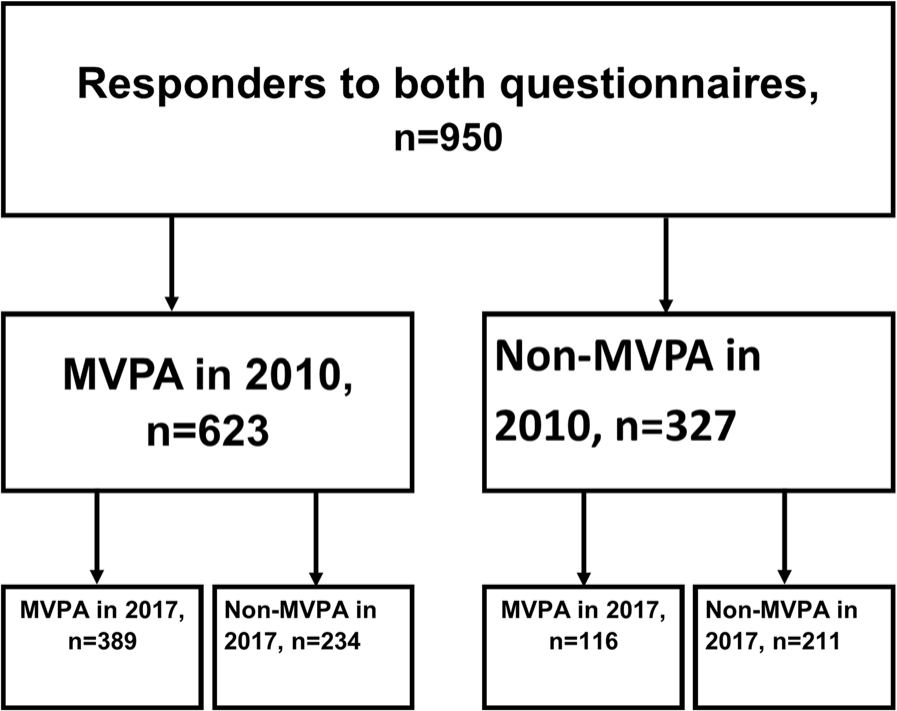
Flowchart of meeting MVPA recommendations or not (non-MVPA) in 2010 and in 2017. MVPA, moderate physical activity and/or vigorous physical activity

Forty-one per cent (*n* = 389) of the patients met MVPA recommendations on both occasions, 66% (*n* = 623) in 2010 and 53% (*n* = 505) in 2017 (Fig. 1). In 2010, 32% reported that they were physically active at a moderate level and 11% at a vigorous level, and 23% fulfilled the criteria by a combination of vigorous and moderate levels of physical activity. The corresponding figures in 2017 were 27, 14, and 12%. A higher proportion of patients who reported having a VPA level in 2010 (*n* = 324) also met MVPA recommendations in 2017 (75%), as compared to those who reported having a MPA level in 2010 (*n* = 299), where 49% met MVPA recommendations in 2017.

Of the 327 patients who did not meet MVPA in 2010, 35% (*n* = 116) improved and met MVPA recommendations in 2017. Of those who met MVPA recommendations in 2010, 38% (*n* = 234) deteriorated and did not meet the MVPA recommendations in 2017 (Fig. 1).

### Factors associated with health enhancing physical activity

Patients who met MVPA recommendations generally had better outcomes in all areas measured at both occasions compared to those who did not meet MVPA recommendations at any occasion. However, there were no significant differences between genders or in the proportions of patients with RF [Additional file 1].

There was a negative association between meeting MVPA recommendations at both occasions and information from the 2010 questionnaire: age, being a smoker, being or obese, the presence of cardiovascular or pulmonary disease, TJC and SJC, PatGA, pain intensity, fatigue, physical function (HAQ), and pain distribution compared with patients who never met the MVPA recommendations. There was a positive association between meeting MVPA recommendations at both occasions and a better QoL in 2010 (EQ-5D), please see Table 2.

**Table 2:** Variables (from 2010) associated with meeting MVPA recommendations on both occasions (*n* = 389) vs. not meeting MVPA at any occasion (*n* = 211) (2010 and 2017). A logistic regression model adjusted for age, gender, and smoking habits

2010		n^a^	OR	95% CI	*p*-value
Age		594	**0.979**	**0.969–0.989**	**< 0.001**
Gender	men	164	1		
	women	430	1.176	0.801–1.726	0.407
Smoking habits	Non smoker	251	1		
	Smoker	92	**0.591**	**0.357–0.980**	**0.041**
	Previous smoker	251	0.743	0.507–1.089	0.128
Disease duration, years		594	0.996	0.953–1.042	0.875
BMI	18.5–24.9, %	262	1		
	< 18.5, %	4	1.050	0.102–10.758	0.968
	25.0–29.9, %	222	**0.503**	**0.334–0.757**	**0.001**
	≥30, %	81	**0.242**	**0.141–0.414**	**< 0.001**
Cardiovasc	no	361	1		
	yes	225	**0.484**	**0.330–0.709**	**< 0.001**
Pulmonary	no	545	1		
	yes	44	**0.483**	**0.256–0.911**	**0.025**
TJC		587	**0.960**	**0.934–0.987**	**0.004**
SJC		587	**0.959**	**0.927–0.992**	**0.015**
PatGA (0–10)		583	**0.883**	**0.819–0.952**	**0.001**
Pain (0–10)		581	**0.908**	**0.846–0.974**	**0.007**
Fatigue (0–10)		585	**0.885**	**0.830–0.944**	**< 0.001**
HAQ (0–3)		589	**0.476**	**0.344–0.658**	**< 0.001**
EQ 5D (0–1)		578	**4.689**	**2.060–10.677**	**< 0.001**
Tender regions (0–18)		594	**0.903**	**0.862–0.946**	**< 0.001**
DMARD	No DMARD	107	1		
	cDMARD^b^	309	0.886	0.549–1.432	0.622
	Biologic^b^	153	0.867	0.504–1.494	0.608
	Only CS	18	0.490	0.174–1.383	0.178

^a^ included in the multivariate analyses, ^b^with and without corticosteroids

*BMI* Body mass index, *TJC* Tender joint count, *SJC* Swollen joint count, *PatGA* Patient Global Assessment, *NRS* Numeric rating scale, *HAQ* Health Assessment Questionnaire, *EQ. 5D* Euroqol 5 Dimensions, *DMARDs* Disease modifying anti-rheumatic drugs, *cDMARDs* Conventional DMARDs, *CS* Corticosteroids

The same factors were associated with a deterioration in physical activity level over time. Patients who fulfilled MVPA recommendations in 2010 but not in 2017 were in 2010 older, more often overweight or obese, had more often cardiovascular and pulmonary comorbidities, reported more pain, worse fatigue and worse physical function and worse pain distribution compared with patients who maintained MVPA recommendations at both time points. There was an inversed relationship with QoL (Table 3).

**Table 3:** Variables (from 2010) associated with a deterioration in in physical activity level between 2010 and 2017 (*n* = 234) vs. meeting MVPA recommendations at both occasions (n = 389). A logistic regression model adjusted for age, gender, and smoking habits

2010		n^a^	OR	95% CI	*p*-value
Age		616	**1.037**	**1.022–1.053**	**< 0.001**
Gender	men	162	1		
	women	454	1.118	0.759–1.647	0.572
Smoking habits	Non smoker	264	1		
	Smoker	93	1.572	0.956–2.587	0.075
	Previous smoker	259	1.263	0.873–1.826	0.216
Disease duration, years		616	0.969	0.928–1.013	0.164
BMI	18.5–24.9, %	290	1		
	< 18.5, %	7	2.361	0.502–11.097	0.277
	25.0–29.9, %	238	**1.702**	**1.180–2.468**	**0.005**
	≥30, %	69	**2.015**	**1.161–3.500**	**0.013**
Cardiovasc	no	375	1		
	yes	235	**1.863**	**1.290–2.691**	**0.001**
Pulmonary	no	565	1		
	yes	47	**2.106**	**1.129–3.927**	**0.019**
TJC		610	1.033	1.006–1.061	0.016
SJC		606	1.025	0.992–1.059	0.138
PatGA (0–10)		611	1.076	0.997–1.160	0.059
Pain (0–10)		607	**1.087**	**1.014–1.164**	**0.018**
Fatigue (0–10)		611	**1.072**	**1.007–1.142**	**0.030**
HAQ (0–3)		611	**1.630**	**1.167–2.278**	**0.004**
EQ 5D (0–1)		605	**0.382**	**0.162–0.899**	**0.028**
Tender regions (0–18)		615	**1.076**	**1.028–1.126**	**0.002**
DMARD	No DMARD	103	1		
	cDMARD^b^	335	1.572	0.960–2.574	0.072
	Biologic^b^	161	1.491	0.858–2.589	0.156
	Only CS	15	1.493	0.472–4.720	0.495

^a^ included in the multivariate analyses, ^b^with and without corticosteroids

*BMI* Body mass index, *TJC* Tender joint count, *SJC* Swollen joint count, *PatGA* Patient Global Assessment, *NRS* Numeric rating scale, *HAQ* Health Assessment Questionnaire, *EQ. 5D* Euroqol 5 Dimensions, *DMARDs* Disease modifying anti-rheumatic drugs, *cDMARDs* Conventional DMARDs, *CS* Corticosteroids

**Table 4:** Variables (from 2010) associated with an improvement in physical activity level between 2010 and 2017 (*n* = 211) vs. not meeting MVPA recommendations at any occasion (n = 116). A logistic regression model adjusted for age, gender, and smoking habits

2010		n^a^	OR	95% CI	*p*-value
Age		322	**0.963**	**0.945–0.981**	**< 0.001**
Gender	men	97	1		
	women	225	1.061	0.628–1.972	0.826
Smoking habits	Non smoker	125	1		
	Smoker	59	0.732	0.379–1.416	0.355
	Previous smoker	138	0.683	0.403–1.156	0.155
Disease duration, years		322	0.991	0.931–1.054	0.764
BMI	18.5–24.9, %	105	1		
	< 18.5, %	3	1.964	0.166–23.159	0.592
	25.0–29.9, %	130	0.829	0.427–1.458	0,516
	≥30, %	62	0.555	0.276–1.116	0.099
Cardiovasc	no	171	1		
	yes	144	0.623	0.371–1.048	0.074
Pulmonary	no	283	1		
	yes	34	1.111	0.523–2.357	0.785
TJC		318	**0.946**	**0.906–0.987**	**0.011**
SJC		314	0.956	0.908–1.006	0.082
PatGA (0–10)		311	0.904	0.812–1.006	0.065
Pain (0–10)		315	0.915	0.826–1.015	0.094
Fatigue (0–10)		317	0.929	0.852–1.012	0.092
HAQ (0–3)		322	0.719	0.463–1.116	0.141
EQ 5D (0–1)		310	**3.773**	**1.059–13.441**	**0.041**
Tender regions (0–18)		322	**0.926**	**0.867–0.988**	**0.020**
DMARD	No DMARD	50	1		
	cDMARD^b^	166	1.180	0.583–2.387	0.645
	Biologic^b^	85	1.401	0.650–3.021	0.390
	Only CS	13	1.263	0.326–4.897	0.735

^a^ included in the multivariate analyses, ^b^with and without corticosteroids

*BMI* Body mass index, *TJC* Tender joint count, *SJC* Swollen joint count, *PatGA* Patient Global Assessment, *NRS* Numeric rating scale, *HAQ* Health Assessment Questionnaire, *EQ. 5D* Euroqol 5 Dimensions, *DMARDs* Disease modifying anti-rheumatic drugs, *cDMARDs* Conventional DMARDs, *CS* Corticosteroids

There was a negative association between patients who improved in physical activity level over time and age, TJC, and pain distribution compared with those who did not meet MVPA recommendations at any occasion. An improvement was also associated with a better QoL (EQ. 5D) in 2010 (Table 4). Overweight or obesity were not statistically significant in the multivariate model, but patients who improved over the 7 years, had in 2010 a lower BMI (mean 25.8 kg/m^2^ SD 4.4 vs. 27.4 kg/m^2^ SD 4.6, *p* = 0.005) compared with those who did not change.

## Discussion

Using a study with a prospective, longitudinal design, we investigated physical activity levels over 7 years in a population-based cohort of patients with established RA. We found that only four out of every ten patients reported having health-enhancing levels of physical activity at both time points. Maintaining recommended levels of physical activity is a challenge to people with RA, and this study searched for characteristic differences between those who stayed active vs. those who did not. We also studied improvement and deterioration in physical activity levels to find important factors to address in the clinic to enhance a healthy lifestyle.

A proportion of the patients who were physically active at baseline maintained this also at follow-up. Patients who reported other unhealthy lifestyle factors such as smoking and overweight, or who reported having cardiovascular or pulmonary comorbidities and worse pain, fatigue, and function in 2010 were less likely to perform health-enhancing physical activity 7 years later. These findings are supported by the findings of earlier cross-sectional studies [12] and the few longitudinal studies published on this subject [16]. Similar factors were of importance when studying those who improved or deteriorated in physical activity level over 7 years. This indicates a need not only to design interventions for improvement of physical activity levels but also to screen for other lifestyle-related factors and design interventions addressing them as well as addressing high levels of pain and fatigue.

Only four out of every ten patients maintained health-enhancing physical activity over time, and one out of every ten patients improved in physical activity level, which may indicate that the healthcare sector has not been successful in screening and coaching a healthy lifestyle. Our patients had all met at least one physician, either in primary or in secondary care during the period investigated, and most likely other health professionals also. In Sweden, all health professionals are recommended to discuss lifestyle factors at healthcare visits in primary and in secondary care, and more studies on successful interventions are needed. In an earlier study on this cohort of patients, a large proportion of the subjects did not recall any discussions about lifestyle habits during their healthcare visits, and a good proportion were not interested in such discussions [26]. A coordinated intervention to support lifestyle changes needs to be implemented in the healthcare sector, and early interventions appear to be helpful [27]. Such an intervention should be designed in line with behavioral theories, including educational components and a longer follow-up period [28, 29]. Personalized medicine may be one of several ways to help people who are at risk of developing RA to adhere to a healthy lifestyle [30–32].

We found transitions between the physically inactive group and the group classified as meeting health-enhancing physical activity recommendations over the 7 years, in both directions. The use of a cut-off may have contributed to the transition, but it was evident that patients who were active at a vigorous level tended to stay active over the years while a greater proportion of those who reported being active at a moderate level were later categorized as being physically inactive. The importance of vigorous exercise, if feasible, also for patients with chronic inflammatory diseases has been highlighted due to its possible anti-inflammatory effect and positive effect on the cardiac system, reducing the risk of cardiovascular disease [8, 9]. If engaged in vigorous exercise as compared to moderate physical activity, people with rheumatic diseases are more likely to maintain healthy levels of physical activity over time [33], which is supported by our findings. In all regression analyses, older age was associated with a worse outcome in physical activity behavior. These findings might indicate a need for better coaching and adjustment of feasible activities, as patients with RA grow older.

Only a third (35%) of the patients who reported being physically inactive at baseline reported being physically active at a moderate level at follow-up, while 65% remained sedentary. We lack information on possible interventions, but we know from earlier studies that those who reported that they were active at a health-enhancing level in 2017 were more likely to have discussed physical activity with health professionals during a healthcare visit [26]. This probably reflects an interest in adhering to a healthy lifestyle, but not necessarily a successful intervention.

Maintenance of healthy levels of physical activity over time is challenging for patients with an established disease. The patient is not always aware of the positive and harmless effects of moderate or vigorous physical activity [34, 35]. Many patients struggle to find a balance in life; to regain a more normal life and adhere to a healthy lifestyle may be of great value, but also have a negative effect on a person’s quality of life [34].

The study was based on self-reported information, and the low correlation between self-reported data, aerobic capacity, and physical activity measured with accelerometers has been well established [36]. However, the findings agree with earlier self-reported levels of physical activity in this population [12, 16]. We do know from earlier studies that a number of biopsychosocial factors may affect a person’s participation in physical activity, factors that were not available in this analysis. A high degree of disease activity, poor mental health, and patient perceptions of disease are associated with a sedentary lifestyle [16]. Also, fear-avoidance belief [37], self-efficacy, social support, and outcome expectations related to physical activity are of importance for participation in physical activity [12]. Self-reported physical activity, and especially moderate levels of physical activity, may be affected by the season in which it is reported [38], so it may be relevant to mention that both questionnaires were sent out in February/March. Yet another limitation is the lack of information concerning physical activity level during the seven-year time span, changes we have not accounted for could have occurred. In addition, self-reports of swollen and tender joints might be considered a limitation, but earlier studies has shown that these measures can be trusted [39].

## Conclusions

Only four out of every ten patients with established RA maintained the recommended levels of physical activity over a seven-year period. Few improved in physical activity level while a larger proportion deteriorated. Worse health was associated with not maintaining health-enhancing physical activity, and the predictive factors were similar to those associated with worse performance in cross-sectional studies. Other unhealthy lifestyle factors and comorbidities reported had an effect on maintenance of physical activity, which is why coordinated supportive lifestyle interventions based on behavioral theories should be implemented in rheumatology care.

## Supplementary information

**Additional file 1.** Cross-sectional description and comparison between patients fulfilling MVPA recommendations or not (non-MVPA) in 2010 and 2017.

## Data Availability

The datasets used and/or analyzed during the current study are available from the corresponding author on reasonable request.

## Abbreviations

ACR: American College of Rheumatology
BARFOT: Better Anti-Rheumatic PharmacOTherapy
BMI: Body Mass Index
DAS28: 28-joint Disease Activity Score
DMARD: Disease-Modifying Anti-Rheumatic Drug
EQ 5 D: Euroqol 5 Dimensions
HAQ: Health Assessment Questionnaire
MVPA: Moderate-to-Vigorous Physical Activity
NRS: Numerical Rating Scale
PatGA: Patient Global Assessment
RA: Rheumatoid arthritis
RF: Rheumatoid factor
SJC: Swollen Joint Count
TJC: Tender Joint Count
QoL: Quality of Life
