# Supplementary material for: Physical activity in established rheumatoid arthritis and variables associated with maintenance of physical activity over a seven-year period – a longitudinal observational study
Source: BMC Rheumatol. 2020 Oct 8;4:53. doi: 10.1186/s41927-020-00151-6 (PMC7542713; doi:10.1186/s41927-020-00151-6)
Supplement: Supplementary file 1 — Additional file 1. Cross-sectional description and comparison between patients fulfilling MVPA recommendations or not (non-MVPA) in 2010 and 2017. [file 41927_2020_151_MOESM1_ESM.pdf]

Additional file 1. Cross-sectional description and comparison between patients fulfilling MVPA recommendations or not (non-MVPA) in 2010 and 2017.

		2010			2017		
		MVPA Mean (SD)	**Non-MVPA** Mean (SD)	P-value	MVPA Mean (SD)	**Non-MVPA** Mean (SD)	P-value
N		389	211				
Age		59 (13)	63 (13)	**<0.001**			
gender	Women, %	75	68	0.099			
Smoking habits	Non smoker, %	46	35		46	35	
	Smoker, %	14	18	**0.041**	11	14	**0.046**
	Previous smoker,	40	46		43	51	
RF, %		65	66	0.746			
Disease duration, years		8.8 (3.9)	8.9 (3.7)	0.897			
BMI		25.0 (3.9)	27.4 (4.6)	**<0.001**	25.2 (4.8)	27.4 (5.1)	**<0.001**
BMI	<18.5, %	1	1		0	2	
	18.5-24.9, %	54	30		55	31	
	25.0-29.9, %	36	45	**<0.001**	34	41	**<0.001**
	≥30, %	9	24		11	26	
TJC		4.7 (5.8)	6.4 (6.9)	**0.003**	4.2 (5.2)	5.9 (7.4)	**0.002**
SJC		3.1 (4.8)	4.2 (5.4)	**0.017**	2.3 (3.7)	3.8 (5.8)	**0.001**
PatGA NRS (0-10)		2.7 (2.2)	3.3 (2.4)	**0.001**	2.5 (2.1)	3.6 (2.7)	**<0.001**
Pain NRS (0-10)		3.0 (2.5)	3.6 (2.6)	**0.009**	3.0 (2.4)	4.2 (2.7)	**<0.001**
Fatigue NRS (0-10)		3.8 (2.8)	4.6 (2.8)	**0.001**	3.6 (2.8)	4.8 (2.9)	**<0.001**
HAQ		0.42 (0.49)	0.66 (0.64)	**<0.001**	0.43 (0.46)	0.84 (0.76)	**<0.001**
EQ5D		0.77 (0.20)	0.70 (0.24)	**<0.001**	0.77 (0.18)	0.64 (0.30)	**<0.001**
Tender regions		3.7 (3.5)	5.1 (4.2)	**<0.001**	3.5 (3.4)	4.6 (4.1)	**0.001**
Cardiovascular disease, %		31	53	**<0.001**	45	55	**0.013**
Pulmonary disease, %		6	12	**0.015**	10	14	0.114
DMARD	No DMARD, %	19	17		23	26	
	cDMARD*	52	54	0.573	45	43	**0.014**
	bDMARD*	26	25		29	23	
	Only corticosteroids*	3	4		3	8	

BMI, body mass index; TJC, tender joint count; SJC, swollen joint count; PatGA, Patient Global Assessment; NRS, numeric rating scale; HAQ, Health Assessment Questionnaire; EQ5D, Euroqol 5 Dimensions; DMARDs, disease modifying anti-rheumatic drugs; cDMARDs, conventional DMARDs; CS, corticosteroids.
